# Incidence of childhood pneumonia: facility-based surveillance estimate compared to measured incidence in a South African birth cohort study

**DOI:** 10.1136/bmjopen-2015-009111

**Published:** 2015-12-18

**Authors:** David M le Roux, Landon Myer, Mark P Nicol, Heather J Zar

**Affiliations:** 1 Department of Paediatrics and Child Health, Red Cross War Memorial Children's Hospital and MRC Unit on Child and Adolescent Health, University of Cape Town, Cape Town, Western Cape, South Africa; 2 Department of Paediatrics, New Somerset Hospital, Cape Town, Western Cape, South Africa; 3 Division of Epidemiology and Biostatistics, School of Public Health and Family Medicine, University of Cape Town, Cape Town, Western Cape, South Africa; 4 Division of Medical Microbiology, University of Cape Town, Cape Town, Western Cape, South Africa

**Keywords:** EPIDEMIOLOGY

## Abstract

**Background:**

Pneumonia is the leading cause of childhood mortality and a major contributor to childhood morbidity, but accurate measurement of pneumonia incidence is challenging. We compared pneumonia incidence using a facility-based surveillance system to estimates from a cohort study conducted contemporaneously in the same community in Cape Town, South Africa.

**Methods:**

A surveillance system was developed in six public sector primary care clinics and in a regional referral hospital, to detect childhood pneumonia cases. Nurses recorded all children presenting to facilities who met WHO case definitions of pneumonia, and hospital records were reviewed. Estimates of pneumonia incidence and severity were compared with incidence rates based on active surveillance in the Drakenstein Child Health Study.

**Results:**

From June 2012 until September 2013, the surveillance system detected 306 pneumonia episodes in children under 1 year of age, an incidence of 0.20 episodes/child-year (e/cy) (95% CI 0.17 to 0.22 e/cy). The incidence in the cohort study from the same period was 0.27 e/cy (95% CI 0.23 to 0.32 e/cy). Pneumonia incidence in the surveillance system was almost 30% lower than in the birth cohort; incidence rate ratio 0.72 (95% CI 0.58 to 0.89). In the surveillance system, 18% were severe pneumonia cases, compared to 23% in the birth cohort, rate ratio 0.81 (95% CI 0.55 to 1.18).

**Conclusions:**

In this setting, facility-based pneumonia surveillance detected fewer cases of pneumonia, and fewer severe cases, compared to the corresponding cohort study. Facility pneumonia surveillance using data collected by local healthcare workers provides a useful estimate of the epidemiology of childhood pneumonia but may underestimate incidence and severity.

**Strengths and limitations of this study**

- Direct comparison of two different methods are used for estimating incidence of childhood pneumonia (facility-based pneumonia surveillance, using routine health workers and official census population figures, compared to incidence measured by trained research workers in a birth cohort).
- The study gives an approximation of the extent to which the pneumonia surveillance system underestimated the incidence of pneumonia.
- The study gives explanation of possible reasons why the surveillance system underestimated the incidence of pneumonia.
- Surveillance data is reported for a relatively short time period: only 16 months (1 full pneumonia season).
- Only infants <1 year of age are included in analysis as too few events had occurred in older children in the birth cohort to enable comparison; this may limit extrapolation to older children.

## Introduction

Pneumonia is the largest single cause of child mortality outside of the neonatal period, accounting for 15% of the approximately 6.3 million global child deaths each year.^1^ ^2^ Pneumonia is also a major cause of childhood morbidity with approximately 120 million episodes of pneumonia occurring annually.^3^ Up to 50% of consultations at healthcare facilities for sick children in low and middle-income countries (LMICs) are due to acute respiratory infections.^4^ Accurate measurement of the incidence of childhood pneumonia is important for allocation of resources, measurement of the impact of interventions such as new vaccines, identifying risk factors and for health system planning.

Estimating the incidence of pneumonia in LMICs can be challenging, with widely varying estimates.^5^ Many studies report incidence of hospitalisation^6^ or of radiologically confirmed^7^ pneumonia, and do not quantify ambulatory events or events without radiological confirmation. Some studies have reported population-level incidence estimates using WHO clinical case definitions in children aged under-1 and under-5 years (under-5), but the incidence estimates are highly variable, reflecting large regional variations in pneumonia incidence.^8^ Most population-level estimates of pneumonia incidence are extrapolated from national under-5 mortality estimates, prevalence of known pneumonia risk factors and likely proportion of deaths due to pneumonia.^5^ From the estimated number of pneumonia deaths, the number of severe pneumonia admissions and ambulatory pneumonia cases can be back calculated.^9^ A recent modelled estimate of pneumonia incidence for children younger than 5 years of age in South Africa was 0·14 episodes/child-year.^10^ However, reliance on modelled estimates of pneumonia incidence can be problematic as the pneumonia incidence models do not necessarily account for local high-prevalence risk factors (such as HIV infection) or changes to healthcare (such as introduction of 13-valent pneumococcal conjugate vaccine, PCV13).

Analysis of routinely collected health systems data provides numbers of childhood pneumonia cases, and may be useful for defining the burden of disease in a district, but calculation of pneumonia incidence based on health service data is complicated by several challenges. These include well-documented concerns with the quality of routine health statistics in many settings, variations in health-seeking behaviours and difficulties in deriving accurate denominators for incidence estimation.^4^ As a result of these concerns, the most reliable method of determining childhood pneumonia incidence is through prospective studies with active pneumonia case finding, independent objective verification of clinical signs of pneumonia and accurate measurement of the person-time at risk throughout follow-up.^11^

The Drakenstein Child Health Study (DCHS), a birth cohort study to investigate the incidence, aetiology and long-term consequences of childhood pneumonia, is being undertaken in South Africa.^12^ As part of this study, pneumonia cases were actively detected among children enrolled in the cohort. In parallel, a facility-based pneumonia surveillance system was established in the communities from which the birth cohort participants were recruited and followed. We describe the establishment and results of the pneumonia surveillance system, and compare the incidence of pneumonia detected through the surveillance system with that measured in the birth cohort in the first year of life.

## Methods

The study was conducted in a peri-urban setting in the Western Cape province of South Africa, approximately 60 km outside Cape Town. Two separate communities were included in both the birth cohort and surveillance system, including six primary care community clinics and the local secondary hospital that serves as a referral point for primary care services, including pneumonia. In one community, Mbekweni, healthcare was provided at two nurse-run primary healthcare (PHC) clinics. The other community, Paarl East, was served by a central nurse-run PHC clinic and by three satellite PHC clinics. Nurses at these clinics treated paediatric presentations according to Integrated Management of Childhood Illness (IMCI) principles; children who met severity criteria were referred to Paarl Hospital. Clinics were open daily on weekdays; on weekends and after hours, care was provided at Paarl Hospital. The childhood immunisation schedule included vaccinations against pertussis, *Haemophilus influenzae* type b and measles, as well as PCV13 at 6 and 14 weeks, and at 9 months. There is very limited utilisation of private sector healthcare services in these communities; individuals usually make use of a single designated clinic; antibiotics cannot be bought over the counter without a prescription.

### Facility-based pneumonia surveillance system

A pneumonia surveillance system was established in the six clinics in the study area. Meetings and training sessions were held with the nursing staff at each clinic to train staff on WHO clinical case definitions.^13^ Training sessions included video clips of children demonstrating clinical signs. Nurses at the six clinics were provided with simple data capture sheets to record information regarding each pneumonia episode that met the WHO criteria (cough or difficulty breathing with age-appropriate tachypnoea or lower chest indrawing). Severe pneumonia classification was based on age and presence of clinical signs. For children aged <2 months, clinical signs included respiratory rate >60 breaths/min, severe chest indrawing, nasal flaring, grunting or fever >37.5°C. For children older than 2 months, clinical signs of severe pneumonia were lower chest wall indrawing, stridor in a calm child, or any general danger sign.^13^ Children older than 2 months with lower chest wall indrawing but no other criteria for severity were retrospectively reclassified as ‘pneumonia’ according to revised 2014 WHO case definitions.^13^ ^14^ The study doctor visited each clinic monthly, and provided on-site refresher training to the nurses. In addition, a local field worker visited each clinic weekly to collect data capture sheets, reinforce the study aims and to encourage the nurses to continue with the surveillance. At Paarl Hospital, data on pneumonia episodes were abstracted from hospital folders of children who met the case definitions and were resident in the catchment area. The child's folder number, date of birth and area of residence were recorded to prevent double counting of pneumonia cases referred from a local clinic to the hospital. Facility-based pneumonia surveillance was conducted from June 2012 to September 2013 in the six community clinics and at Paarl Hospital.

### Measurement of pneumonia incidence in the DCHS

The DCHS is a prospective birth cohort with the primary aim to investigate the incidence, aetiology and long-term consequences of childhood pneumonia. Pregnant women were enrolled during antenatal care and their children were enrolled from birth. Regular scheduled follow-up visits occurred through the first year of life at primary care clinics or at Paarl Hospital.^12^

The method of pneumonia case detection in the surveillance system differed from that of the birth cohort (table 1). In the birth cohort, pneumonia events were identified in real time. Active surveillance for pneumonia was performed by nurses at the six participating clinics, who referred birth cohort participants with respiratory symptoms to the research study nurses for assessment, or by directly contacting the study staff through a 24 h study cell phone. WHO pneumonia case definitions were applied^14^; study nurses were trained in respiratory examination of children, and had regular competency assessments. As the mothers were interviewed frequently through their child's first year of life, and study staff always enquired about previous respiratory events, it was possible to retrospectively capture pneumonia events occurring at other facilities or outside the area; information was obtained by review of medical records at the admitting facility.^15^

**Table 1:** Comparison of methods of pneumonia surveillance system and birth cohort

	Facility-based pneumonia surveillance system	DCHS Birth cohort^15^
Time period	June 2012–September 2013 (calendar-year incidence calculation: 1 September 2012–31 August 2013	29 May 2012–31 May 2014
Study population	All children <5 years resident in the catchment area; incidence calculated for children <1 year of age	Birth cohort participants in first year of life; 697 infants born between 29 May 2012 and 31 May 2014
Estimation of person-time at risk	Estimated 1292 children <1 year of age from Department of Health population statistics	Calculated for each individual from date of birth until death/disenrolment/first birthday
Method of pneumonia case detection	Clinics: PHC nurse identification of IMCI pneumonia at each facility, and basic data collection form completed Hospital: record review of children resident in catchment area attending hospital	Examination by trained study nurse; real-time interview of mother; record review of medical notes Retrospective inclusion of cases: if medical records indicated that they met WHO pneumonia criteria

DCHS, Drakenstein Child Health Study; ICMI, Integrated Management of Childhood Illness; PHC, primary healthcare.

### Analysis and statistics

In analysis, continuous variables were described as medians (with IQRs) and categorical variables as proportions with 95 CIs. When using health facility surveillance data, incidence was calculated as the number of pneumonia cases occurring over a continuous 12-month period, using the midpoint population of children under 5 years of age within the catchment area (as reported by the Western Cape Department of Health) as denominator. Incidence rate ratios and risk ratios were calculated with CI based on the Poisson distribution, and proportions were compared with χ^2^ tests. All p values are two tailed, with α set at 0.05. In comparisons of seasonal incidence, we categorised December to February as summer, March to May as autumn, June to August as winter and September to November as spring.

## Results

**Figure 1:**
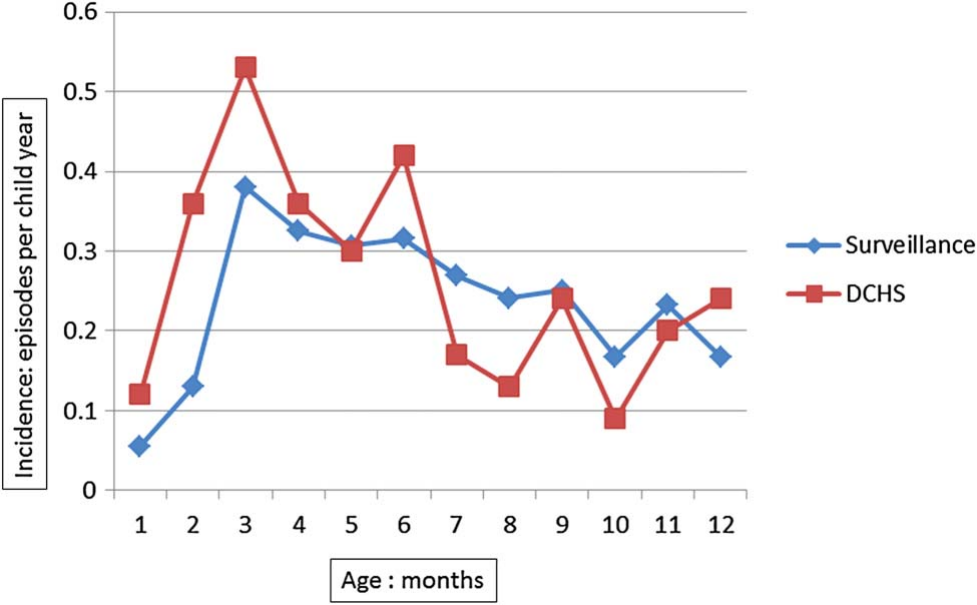
Incidence of pneumonia in the first year of life by age, comparing surveillance system to birth cohort.

From 1 June 2012 to 30 September 2013, 306 pneumonia events were detected by the surveillance system in children under 1 year of age, of which 56 (18%) were severe pneumonia cases. The median age of pneumonia diagnosis was 25 weeks, (IQR=15–36 weeks). The number of pneumonia cases was lowest in the neonatal period, while the highest burden was in the third month of life (figure 1). In 102 pneumonia events (33%), the child was taken directly to the hospital. Of the 204 pneumonia cases identified at the local clinic, 41 (20%) were referred to Paarl Hospital. Overall, 52 infants under 1 year of age (17%) were admitted to hospital. Younger infants were more likely to be admitted to hospital: 21% of infants under 6 months were hospitalised, compared to 13% of infants aged between 6 months and 1 year of age, p=0.008.

There were 1292 infants under 1 year living in the catchment area of the six clinics at the midpoint of 2013. For the first full year for which data were available (1 August 2012 until 31 July 2013), the incidence of pneumonia in children in the first year of life was 0.20 episodes/child-year (95% CI 0.17 to 0.22) (table 2).

**Table 2:** Results of pneumonia surveillance system, compared to birth cohort

	Facility-based pneumonia surveillance system	DCHS Birth cohort^15^	Comparison (comparing surveillance system to birth cohort)
Incidence: episodes/child year	**0.20 (0.17–0.22)**	**0.27 (0.23–0.32)**	**IRR 0.72 (0.58–0.89; p=0.002)**
Admitted	**52/306 (17%)**	**55/141 (39%)**	**RR 0.44 (0.32–0.60; p<0.001)**
Severe pneumonia*	56/306 (18%)	32/141 (23%)	RR 0.81 (0.55–1.18; p=0.28)
Male gender	Pneumonia events in males: 194/306=63%	Events in males: 97/141 (69%)	RR 0.92 (0.80–1.06; p=0.27)
Symptoms			
Fever	**87/306 (28%)**	**78/141 (55%)**	**RR 0.51 (0.41–0.65; p<0.001)**
Wheeze	**93/306 (30%)**	**92/141 (65%)**	**RR 0.47 (0.38–0.57; p<0.001)**
Lower chest indrawing	**82/306 (27%)**	**81/141 (57%)**	**RR 0.47 (0.37–0.59; p<0.001)**
Seasons	(n=254 in 1 calendar-year)	(n=141)	
Summer	35 (14%	14 (10%)	p=0.26
Autumn	71 (28%)	39 (28%)	p=0.95
Winter	92 (36%)	47 (33%)	p=0.56
Spring	56 (22%)	41 (29%)	p=0.12

Bold typeface indicates statistically significant results.

*Criteria for severity as per revised 2014 WHO pneumonia criteria.

DCHS, Drakenstein Child Health Study; IRR, incidence rate ratio; RR, risk ratio.

In the DCHS birth cohort, enrolment of pregnant women began in March 2012, and the first babies were born in May 2012.^12^ In the first year of life, 697 children accrued 513 child-years of follow-up. There were 141 pneumonia cases, with an incidence of 0.27 episodes/child-year (95% CI 0.23 to 0.32); 32 (23%) of the pneumonia cases were severe, and 55 (39%) were admitted to hospital (table 2).^15^ One hundred and twenty-nine cases were identified in real time; 12 cases were identified retrospectively from hospital or clinic records.

The incidence of pneumonia was statistically significantly lower in the surveillance system than in the birth cohort, incidence rate ratio 0.72, 95% CI 0.58 to 0.89. This was most apparent in the first 6 months of life (figure 1).

In addition, the proportion of children in the first year of life who were admitted to hospital was statistically significantly lower as measured by the surveillance system (17% vs 39%, p<0.001). Seasonal variation and gender distribution of pneumonia cases were similar between the birth cohort and the surveillance system, with more cases identified in males, and more cases in autumn and winter than in summer and spring (table 2). When a sample of birth cohort pneumonia events were traced in surveillance system database, 29% had not been identified or reported by the surveillance system.

## Discussion

These results demonstrate a substantial incidence of pneumonia in children under 1 year in this setting, observed in both the facility surveillance system and the birth cohort. This is despite a good PHC system and comprehensive vaccination programme. However, incidence of pneumonia as estimated by the surveillance system was somewhat lower than the incidence measured in the birth cohort in the same communities over a similar time period.

There are several reasons why the incidence estimate in the surveillance system was lower than in the birth cohort. Under-reporting of cases at some clinics is possible; some nurses were initially reluctant to complete the surveillance form, and admitted that they forgot about it if they were not visited and reminded. However, as the seasonal variation of pneumonia cases looks similar in the birth cohort, it is unlikely that there was significant decline in pneumonia reporting over the course of the year. Underdetection of pneumonia cases is also possible. Nurses in the clinics were all IMCI-trained, and ongoing training and support was provided, but no formal skills assessment or objective verification of clinical signs was performed. Thus some misdiagnosis of pneumonia cases, and misclassification of severity of disease, is possible. Nurses working in busy clinics may have had less time for accurate completion of the surveillance forms than the study staff on the birth cohort. This may also have contributed to underdetection of severe pneumonia in the surveillance system compared to the birth cohort. Furthermore, children resident in the area who did not attend the local health facility would not have been included in the incidence calculation. There is evidence from a multinational community-based household survey of respiratory illness that up to 50% of children under 5 years of age with respiratory symptoms are not taken to a formal health facility.^16^

It is possible that the DCHS birth cohort overestimated the incidence of pneumonia in these communities, and it is possible that birth cohort participants were more likely to seek care at local clinics than non-participants. However, misclassification bias due to overdiagnosis is unlikely, as the WHO standardised criteria were applied, and birth cohort staff members were well-trained and supervised, and completed regular competency assessments. However, a selection bias may have been introduced if the women who were enrolled in the birth cohort were significantly more at risk for pneumonia than the rest of the local population. This is unlikely, as enrolment for the birth cohort occurred between 20 and 28 weeks gestation, and women were excluded if they had no fixed abode or could not commit to stay in the area. Thus the birth cohort tended to enrol women from comparatively stable social backgrounds. Thus the birth cohort participants may have been at lower risk of pneumonia than many of the other children in these communities.

Accurate estimation of pneumonia incidence is not possible when the person-time at risk is not known. For the surveillance system, the official Department of Health population statistics were used; but if this population was overestimated, then the estimated incidence would be falsely low. This is a major strength of a birth cohort, where person-time at risk is accurately known for each individual, and all pneumonia events can be recorded, even if identified retrospectively. The DCHS birth cohort required intensive follow-up and active pneumonia case detection, which was resource intense to establish and maintain. It provided an accurate measure of the incidence of pneumonia in this area. The parallel surveillance system was comparatively easier to establish, as clinic staff were already deployed and trained, and all that was required was to request them to complete a single extra data capture form. However, the clinical detail that was available on the surveillance form was limited, and there was no mechanism to control the quality of the data.

This study is unique in that it reports the ‘real world’ pneumonia counts, as diagnosed by local healthcare workers and converted to incidence estimates using official census data; this incidence estimate is compared to an incidence calculation based on active case finding and accurate assessment of person-time at risk. From these data, it appears that the surveillance system, using routine healthcare providers, seems to have systematically underestimated the incidence of pneumonia by about 30% in children under 1 year of age in these communities; and that some of the clinical details and severity assessments also appear to be under-reported, and may be less reliable than those reported in the birth cohort. The lack of detail of clinical signs of pneumonia may make retrospective assessment and regrading of pneumonia severity especially challenging.

Establishment and maintenance of a clinic-based pneumonia surveillance system is feasible in a peri-urban area in a middle income country, and has provided a valuable estimate of the incidence of childhood pneumonia as measured by health services in this area, and of the age distribution of the pneumonia cases. The results of the pneumonia surveillance system has informed the policies, procedures and resource allocation of the DCHS birth cohort study; the DCHS will continue to follow the children in these communities for 5 years, and will determine the accuracy of the surveillance system estimates in children aged 1–5 years. These data will enable researchers to allow for a correction factor in pneumonia incidence calculations, or to quantify the potential for underdetection or misclassification that is possible when using routine health facility staff outside of a clinical trial.
